# Immunohistochemical detection of Polo-like kinase-1 (PLK1) in primary breast cancer is associated with *TP53 *mutation and poor clinical outcome

**DOI:** 10.1186/bcr3136

**Published:** 2012-03-08

**Authors:** Sharon I King, Colin A Purdie, Susan E Bray, Philip R Quinlan, Lee B Jordan, Alastair M Thompson, David W Meek

**Affiliations:** 1 Division of Cancer Research, Medical Research Institute, Ninewells Hospital and Medical School, University of Dundee, Dundee DD1 9SY, UK

## Abstract

**Introduction:**

Polo-like kinase-1 (PLK1) is a crucial driver of cell cycle progression and its down-regulation plays an important checkpoint role in response to DNA damage. Mechanistically, this is mediated by p53 which represses *PLK1 *expression through chromatin remodelling. Consistent with this model, cultured cells lacking p53 fail to repress *PLK1 *expression. This study examined PLK1 expression, p53 mutation and clinical outcome in breast cancer.

**Methods:**

Immunohistochemistry was performed using antibodies to PLK1, MDM2 and Ki67 on Tissue Micro-Array (TMA) slides of a cohort of 215 primary breast cancers. The *TP53 *gene (encoding p53) was sequenced in all tumour samples. Protein expression scored using the "Quickscore" method was compared with clinical and pathological data, including survival.

**Results:**

Staining of PLK1 was observed in 11% of primary breast tumours and was significantly associated with the presence of *TP53 *mutation (*P *= 0.0063). Moreover, patients with both PLK1 expression and *TP53 *mutation showed a significantly worse survival than those with either PLK1 expression or *TP53 *mutation alone. There was also a close association of elevated PLK1 with triple negative tumours, considered to be poor prognosis breast cancers that generally harbour *TP53 *mutation. Further association was observed between elevated PLK1 levels and the major p53 negative regulator, MDM2.

**Conclusions:**

The significant association between elevated PLK1 and *TP53 *mutation in women with breast cancer is consistent with escape from repression of *PLK1 *expression by mutant p53. Tumours expressing elevated PLK1, but lacking functional p53, may be potential targets for novel anti-PLK1-targeted drugs.

## Introduction

Breast cancer remains the most common cancer in women in the western world. The genetic and molecular changes underlying the disease are complex. However, understanding the nature of these changes, and their potential for therapeutic exploitation, presents enormous opportunities for individualised approaches to treatment. To explore these possibilities, there is a need to determine whether mechanistic events established in cultured cell systems, which are thought to drive cancer initiation and/or progression, do indeed underlie the development of the disease in the patients. The testing of clinical material, with focus on the presence or absence of key cancer-associated proteins, should help provide such supporting evidence and identify relevant new candidate markers and therapeutic targets.

The p53 tumour suppressor is a short-lived transcription factor that plays a critical role in eliminating tumour cells by coordinating changes in gene expression, leading to cell cycle arrest, senescence or apoptosis [1-3]. p53 regulates the expression of many genes and, accordingly, loss of p53 function during tumour development can have wide-ranging consequences for the pathology of the tumour cells [2,4]. Most p53 target genes are actually repressed, as opposed to transactivated, by p53 [1], with the outcome that loss of p53 function may lead to dysregulated or even unrestricted levels of oncogenic proteins. Effective transrepression is, therefore, fundamental to p53-mediated tumour suppression, and potentially to clinical outcome. Indeed, mutation of the gene encoding p53 (*TP53*) is associated with worse survival in breast cancer (for example, see [5]).

The protein kinase PLK1 plays a pivotal role in the maturation of centrosomes, entry into M phase, spindle formation and cytokinesis [6-8]. Ectopic expression of PLK1 in cultured cells is oncogenic [9] and, consistent with this observation, elevated PLK1 levels occur in various human tumour types [10-20], including breast cancers where it is associated with aggressive characteristics, such as vascular invasion, markers of proliferative activity and lack of detectable estrogen receptor [17,21]. PLK1 is down-regulated by p53 as part of the G2/M cell cycle checkpoint [22-29] and we recently established that this occurs mainly through p53-dependent repression of *PLK1 *expression [30]. Consistent with this observation, we and others have also shown that p53-null cells are unable to down-regulate PLK1 levels in response to clinically-relevant genotoxic drugs [26,30]. These data suggest the possibility that tumours lacking functional p53 are likely to have dysregulated PLK1 levels and that these, in turn, may contribute towards the development of malignancy. Recent evidence has also suggested that the viability of stressed cells that lack p53 may become dependent upon PLK1 [26]. Given that PLK1 is widely considered to be a potential therapeutic target, and that several PLK1 inhibitors are currently undergoing clinical trials [7], targeting of cancers lacking p53 with inhibitors of PLK1 could provide an effective tailored therapeutic strategy. However, the relationship between PLK1 levels and the status of the p53 pathway in tumours needs to be established.

In the present study, we confirm that a proportion of breast tumours are positive for PLK1 protein expression as judged by immunohistochemical staining. Strikingly, we find a statistically significant correlation between the detection of PLK1 and the acquisition of mutation(s) in the *TP53 *gene. Additionally, we find an association between elevated PLK1 levels and increased levels of the major p53 negative regulator, MDM2. These findings support the established *in vitro *molecular model for *PLK1 *repression by p53 [30] and suggest that elevated PLK1 levels in tumours arise, at least in part, through the absence of functional p53. To our knowledge, this is the first demonstration of such an association. From a clinical perspective, we find that patients with *TP53 *mutation and detectable PLK1 show reduced survival and are more likely to have a triple negative genotype. Our data also highlight the possibility that PLK1-targeted therapy may be a useful option for tumours lacking functional p53.

## Materials and methods

### Patients

The cohort used in this study (TMA24) has been reported previously [31] and comprises 215 unselected pre- and post-menopausal women (aged 28 to 89; median 62 years) with primary, previously untreated breast cancer, who were seen and treated at Tayside University Hospitals, Scotland from 1997 to 2002. Samples were obtained at the time of surgery and only where sufficient cancer material was available. Informed consent was obtained from each patient prior to tissue acquisition and before surgery was carried out. Tumour samples from these patients were constructed on tissue microarray (TMA) slides as described previously following review of tissue blocks by specialist breast pathologists [31]; (the TMAs were constructed from six cores per tumour from across the tumour, not restricted to the edge of the cancer. The values stated are the mean across all six cores). Ethics approval was given by the Tayside Research and Ethics Committee (Ref. 07/S1402/90). The PLK1 analysis was conducted after a median of eight years follow-up during which some patients had recurred or died. Standard adjuvant treatment was used: for those patients with ER-positive tumours tamoxifen or aromatase inhibitors (for post-menopausal women only) and for node-positive patients FEC75 chemotherapy; no Herceptin was used on this cohort.

### Western blotting analysis

This was carried out as reported previously [30].

### Immunohistochemistry (IHC)

Sections from the TMA block (nominally four microns thick) were cut onto Superfrost^® ^coated glass slides (VWR International Ltd., Dublin, Ireland) and dried for one hour at 60°C before being de-paraffinised in Histoclear (National Diagnostics, Altanta, GA, USA) and then rehydrated through a graded alcohol series. A total of 10 mM Citric acid buffer was used as the standard microwave-based antigen retrieval method. Sections were boiled in a pressure cooker for 10 minutes before being immunostained on a DAKO autostainer (Ely, Cambridgeshire, UK) using Vectastain^® ^ABC kits (Vector Labs, Burlingame, CA, USA) according to the manufacturer's protocol. Briefly, sections were blocked in normal goat serum containing 10% (v/v) from stock avidin solution (Vector Labs) for 20 minutes followed by a 1 h incubation with Polo-like Kinase-1 primary antibody (monoclonal antibody 208G4 from Cell Signaling, Boston, MA, USA) at a dilution of 1 in 25 of the material provided by the supplier), including 10% (v/v) from stock biotin solution (Vector Labs) to reduce non-specific background staining. Sections were then incubated with biotinylated anti-rabbit secondary antibody for 30 minutes followed by Vectastain^® ^Elite ABC reagent for another 30 minutes. Liquid Diaminobenzidine (DAB) (DAKO) was used as a chromogenic agent for five minutes and sections were counterstained with Mayer's haematoxylin. In between each immunostaining step, slides were washed briefly in Tris buffered saline (TBS), pH 7.6. Sections known to stain positively were included in each batch and negative controls were prepared by replacing the primary antibody with TBS buffer. Antibodies specific for other proteins used in the study have been reported elsewhere [31].

### TP53 *mutation status *

The mutation status of the *TP53 *gene was assessed as described previously in the tissue samples using the Roche *p53 *Amplichip research test (Roche Molecular Systems, Pleasanton, CA, USA); these data and the detailed methodology are given in reference [32].

### Scoring

TMA scoring for PLK1 was carried out by a specialist breast pathologist (CAP) using a Nikon Eclipse E600 light microscope (Amstelveen, The Netherlands), Aperio, Scanscope XT and Spectrum plus (version 9.1, Vista, CA, USA). Scoring was semi quantitative using the Quickscore method [33] for the intensity and proportion of cells stained for the PLK1 antibody. Scores 0 to 3 indicated the intensity (0 = no staining, 1 = light staining, 2 = moderate staining, 3 = strong staining) whilst scores 1 to 6 represented the proportion of staining (1 = 0 to 4%, 2 = 5 to 20%, 3 = 21 to 40%, 4 = 41 to 60%, 5 = 61 to 80%, 6 = 81 to 100%). Multiplication of each score obtained an overall result of 0 to 18. Cores taken from a plug of MCF7 cells were used as a positive control.

### Statistics

**Figure 1:**
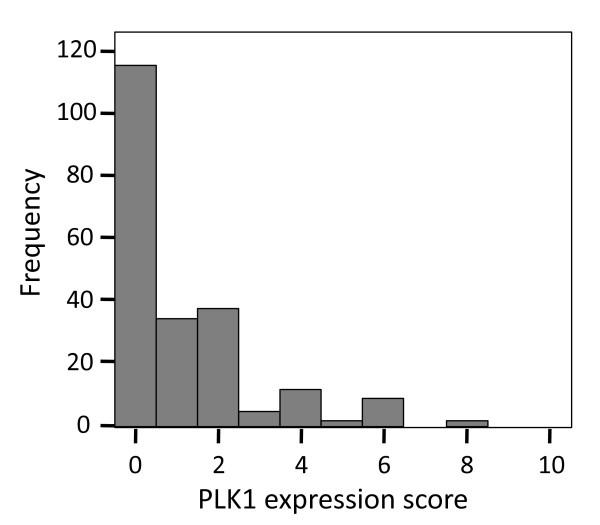
**Histogram of PLK1 staining**. Distribution of frequency of patients against immunohistochemical score of PLK1 staining. Dip test statistic = 0.087, alpha = 0.036, *P *< 0.01, identifying a score of 3 as a cut off.

TMA scoring was analysed to determine appropriate cut-offs for positive PLK1 or negative staining. The frequency of each score was plotted as a histogram and passed through a Dip test to determine the modality of the data. This resulted in a separation between negative (0 to 3) and positive (4 to 18) stained cases (Figure 1).

### Data analysis

The scoring data were automatically extracted from the Aperio Digital Pathology system and combined with the pathology and clinical data from the locally developed digital pathology database. This resulting data set was then analysed using an internally developed automated data analysis system, INSPIRE [34]. For this project, INSPIRE was employed to exhaustively test the results of the PLK1 staining against the standard clinical/pathological outcomes and other staining results held within the database using the two-sided Fisher's exact tests and, where appropriate for survival parameters, the Kaplan-Meier method. Results were considered significant at an α level of 5% (*P *≤ 0.05), with anything above 10% (*P *≤ 0.10) considered not significant. Results falling between these two boundaries (0.05 <*P *≤ 0.10) were marked as marginal in the event any warranted further investigation. The data from INSPIRE were then made available to the research team via a Comma Separated Values (CSV) file to allow further manual analysis. Results that were found to be significant were then re-analysed manually with SPSS Statistics (Version 17, IBM Corp., Armonk, NY, USA) to confirm the results found within INSPIRE. Manual analysis, again using SPSS, was also used to selectively combine variables, such as PLK and p53 status.

Survival was calculated from the date of diagnosis until either the last confirmed hospital visit or the date of death. An event was only recorded if there was confirmation of a breast cancer death. All deaths not directly attributable to breast cancer were censored on the date of death. A relapse was defined as clinical, radiological or pathological evidence of disease relapse.

### Multivariate analysis

To clarify the results of univariate analysis, PLK1 was tested for significance within a multivariate environment using the Cox regression with Backward Conditional method to select the most relevant data when predicting survival outcome.

## Results and discussion

### Staining of PLK1 in breast tumour samples

**Figure 2:**
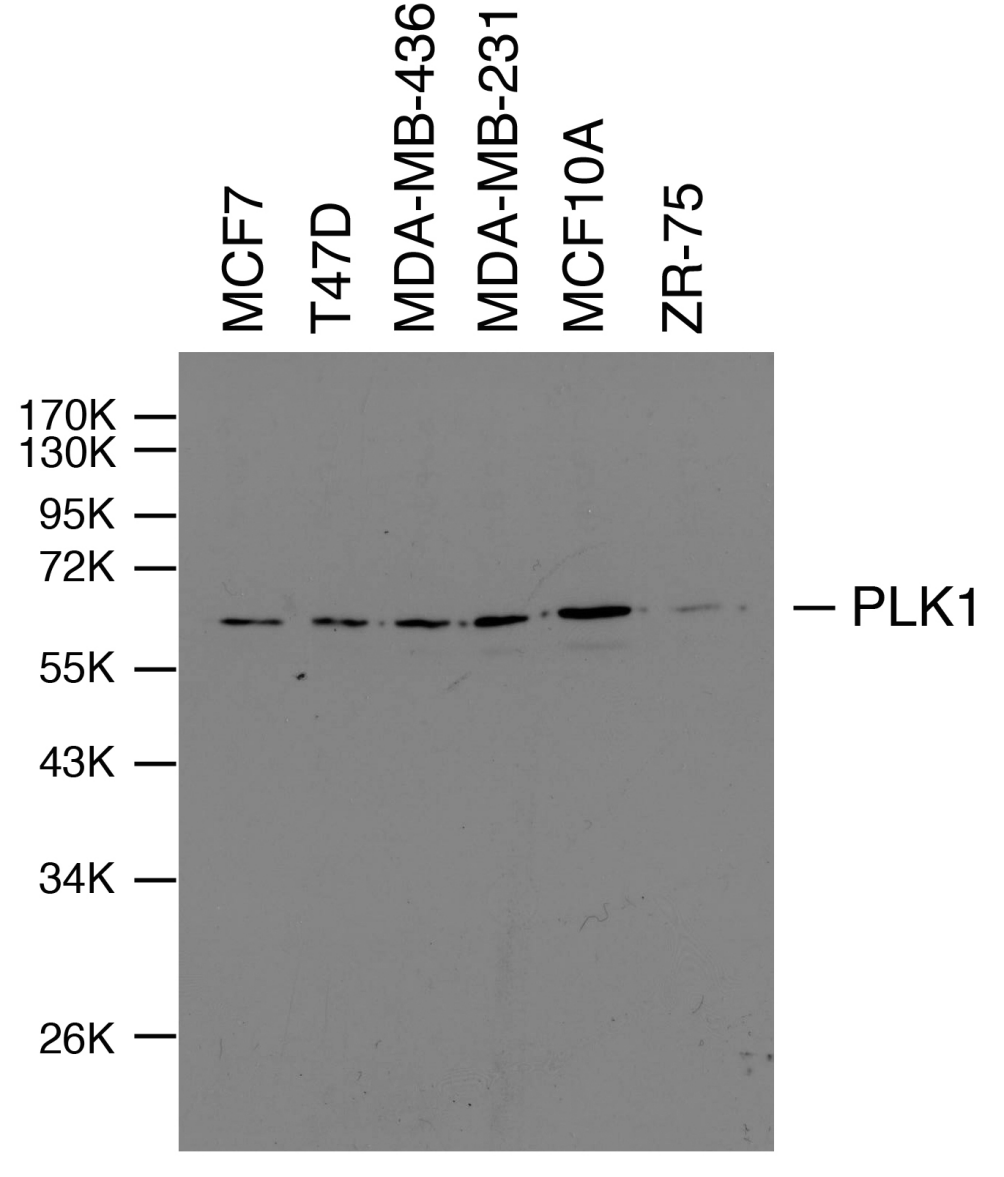
**Anti-PLK1 antibody 208G4 detects only PLK1 in extracts of several breast cancer-derived cell lines**. Cell extracts were prepared by lysing the cells in SDS-sample buffer and the proteins were subsequently resolved by SDS-PAGE. Western blotting analysis reveals a single band of molecular weight 62 kD corresponding to full length PLK1 in each of the cell extracts.

The specificity of the antibody for cell staining was confirmed by Western blotting analysis of cell extracts carried out using the monoclonal antibody, 208G4 (Figure 2). Several different breast cancer-derived cell lines were examined, and were representative of different breast cancer subtypes, p53 wild type/mutant status and the presence or absence of ERα, PR and HER-2. In each case, a single band of expected molecular weight 62 kD (as measured relative to the migration of standards of known molecular weight) was observed corresponding to full length PLK1. These data underpin the specificity of the antibody and validate its application for the immunohistochemical staining of the tumour sections.

**Figure 3:**
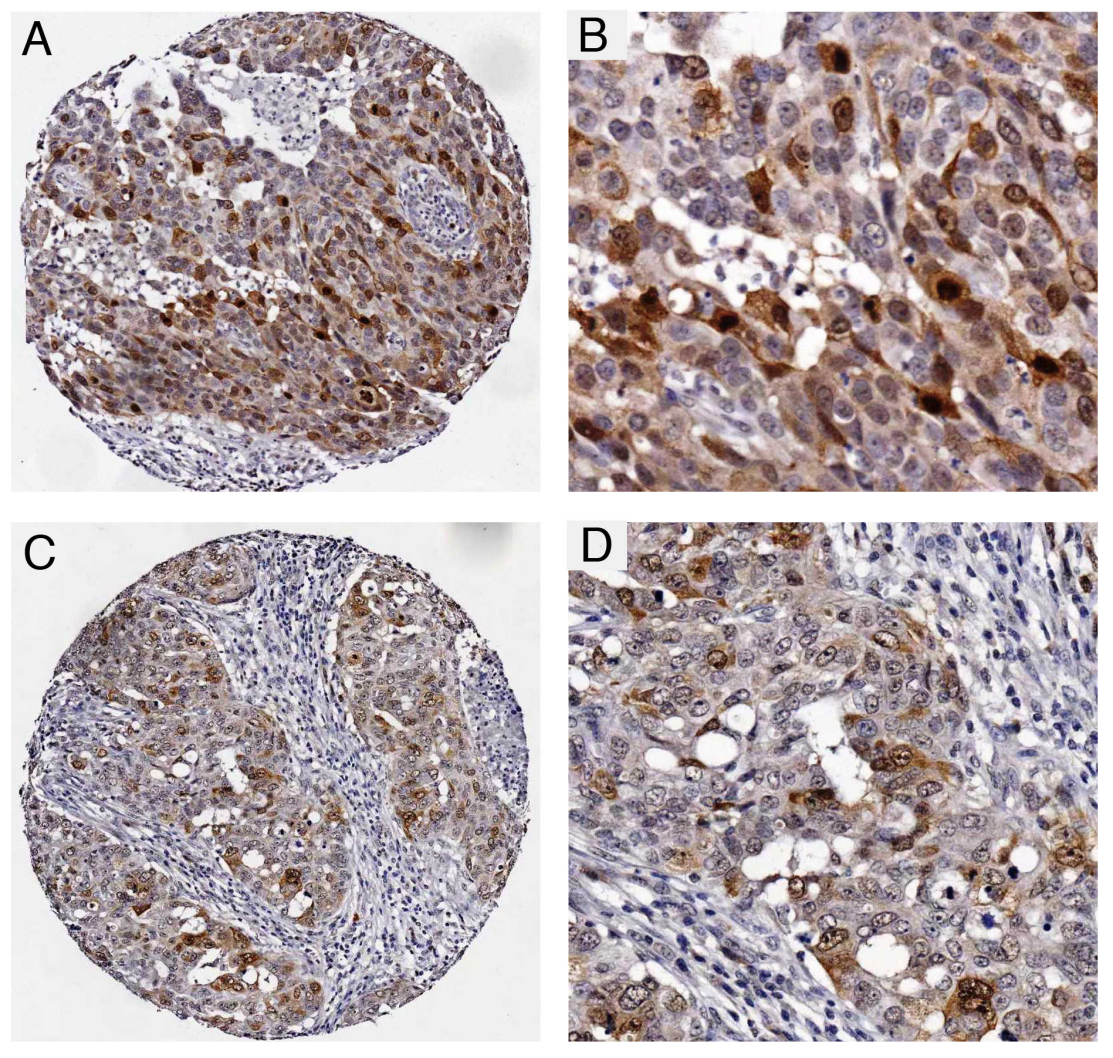
**Photomicrographs of PLK1 immunohistochemistry from tissue microarray**. **A **and **B **Grade 3 invasive ductal carcinoma showing a heterogeneous pattern of nuclear and cytoplasmic staining at low (A) and high (B) power. **C **and **D **grade 3 invasive ductal carcinoma showing similar staining but in fewer cells at low (C) and high (D) power. The staining is highly specific and confined to the carcinoma cells. There is no staining of vascular, stromal or lymphoid cells.

The levels of PLK1 staining in the breast tumour samples varied significantly. A large proportion of samples showed no detectable staining while those that did gave a positive signal (23/215; 11% - see Table 1) varied widely in intensity both from tumour to tumour and, in some cases, within a tumour section, (that is, a small proportion of cells show intense staining as compared with a much lower level of staining in the surrounding cells). Representative examples are shown in Figure 3. These micrographs also indicate that PLK1 staining, where identified, was usually observed in both the nucleus and cytoplasm. Weak or undetectable staining was observed in normal tissue surrounding the tumours. A correlation between PLK1 levels and proliferative activity in cycling cultured cells and in tumours was initially established by Yuan and colleagues [35]. They also noted especially strong staining of mitotic cells, an observation that is consistent with our current knowledge of the cell cycle-dependent regulation of PLK1 expression (that is, only low levels of PLK1 are present in cells in the G1 and S phases as opposed to cells in late G2 and M phase where the levels rise significantly [36-39]). It is, therefore, possible that the variation in staining in the tumour samples could reflect, at least in part, different proliferation rates of the cells. Consistent with this idea we also note that PLK1 was associated with Ki67 expression (a marker for cell proliferation - Table 1). It is also possible that intensely staining cells in any given section are those in G2 or mitosis. Over and above these proposed mechanisms, however, it is likely that cancer-associated changes in the regulation of PLK1 levels additionally contribute to the differences in levels between different tumour samples (see below). Such changes could affect the basal level of expression or the ability to down-regulate PLK1 in response to environmental changes, independently of control mediated by the cell cycle.

**Table 1:** Clinico-pathological characteristics of the patients and the odds ratios

Parameter		PLK1	Staining	*P-*value	Odds Ratio
		Pos (N(%))	Neg (N(%))		
N		23 (10.7)	192 (89.3)	-	-
P53 mutational status	mutant	12 (5.6)	46 (21.4)	0.0063	3.46
	wild type	11 (5.1)	146 (67.9)		
	unknown	0	0		
Histological Grade	1 to 2	2 (1.0)	108 (51.2)	< 0.001	14.18
	3	21 (10.0)	80 (37.9)		
	unknown	0	4		
Survival	dead	9 (4.2)	37 (15.8)	0.02 (Log Rank)	2.318 (HR)
	alive	14 (6.5)	155 (73.5)		
	unknown	0	0		
Menopause	pre-peri	8 (3.9)	42 (20.2)	0.1861	1.96
	post	14 (6.7)	144 (69.2)		
	unknown	1	6		
Lymph Node Status	positive	12 (5.7)	90 (42.7)	0.8257	1.19
	negative	11 (5.2)	98 (46.5)		
	unknown	0	4		
Size	< 2 cm	17 (7.9)	138 (64.2)	1	1.11
	>/ = 2 cm	6 (2.8)	54 (25.1)		
	unknown	0	0		
Luminal A	positive	7 (3.3)	99 (47.1)	0.075	0.42
	negative	15 (7.1)	90 (42.4)		
	unknown	1	3		
Luminal B	positive	2 (1.0)	37 (17.6)	0.382	0.41
	negative	20 (9.5)	152 (71.9)		
	unknown	1	3		
HER2	positive	5 (2.4)	28 (12.9)	0.344	1.76
	negative	17 (8.1)	162 (76.7)		
	unknown	1	2		
ER-alpha	positive	12 (5.6)	151 (70.6)	0.0081	3.46
	negative	11 (5.1)	40 (18.7)		
	unknown	0	1		
PR	positive	10 (4.7)	110 (51.4)	0.266	0.57
	negative	13 (6.1)	81 (37.9)		
	unknown	0	1		
Triple negative	yes	8 (3.8)	25 (11.9)	0.010	3.8
	no	14 (6.7)	164 (77.6)		
	unknown	1	3		
MDM2	positive	10 (4.7)	31 (14.5)	0.004	3.95
	negative	13 (6.1)	159 (74.7)		
	unknown	0	2		
Ki67	positive	9 (4.3)	19 (9.1)	0.0004	6.63
	negative	12 (5.8)	168 (80.8)		
	unknown	1	5		

The Odds ratio determines the likelihood of showing any given characteristic if the tumour is PLK1 positive as opposed to PLK1 negative. Where numbers in individual categories do not add up to 215 this is due, in part, to loss of TMA spot data (tumour cut out, folded spots, low cellularity) from individual patients. *Luminal stains are based on IHC data and are defined as: luminal A, ER^+ ^PR^+ ^HER2^- ^; and luminal B, ER^+ ^PR^- ^HER2^-^

### Relationship of PLK1 expression to *TP53 *status in breast cancer patients

There is a significant association between *TP53 *mutation status and PLK1 levels (Table 1) such that there is a 3.5-fold greater likelihood of having detectable PLK1 staining if the tumour harbours a mutation(s) in the *TP53 *gene. We showed recently that *PLK1 *expression is tightly regulated through p53-mediated transcriptional repression [30]. The association between *PLK1 *expression and *TP53 *mutation is, therefore, consistent with our model [30] and suggests that elevated PLK1 levels are likely to occur, at least in part, through the loss of p53 function. We and others have also shown that p53 is protective against apoptosis induced by down-regulation of PLK1 in cultured cells [26,30,40]. This observation suggests that tumour cells retaining wild type p53 function are likely to be less susceptible to PLK1 inhibitors and, accordingly, that p53 status is a factor that will have to be taken into account when considering the use of PLK1 inhibitors as part of a "personalised medicine" approach. This is especially significant given that breast cancer patients who have mutant p53 are less responsive to conventional chemotherapies [5]. For these patients, PLK1 inhibitors might be particularly relevant.

Mutation of *TP53 *can lead not only to inactivation of p53 function but many mutant p53 proteins can interfere with wild type p53 function in a dominant-negative manner in p53 wild type/mutant heterozygotes (reviewed in [41]). Moreover, certain p53 mutants are thought to acquire new functional properties that can influence gene expression levels in a manner that is unaffected by wild type p53. Many of these mutants have been linked to poor prognosis in patients, and studies with animal models indicate that they are likely to promote tumour progression [41,42]. Our published molecular and cellular analyses suggest that two commonly-occurring tumour-associated "gain-of-function" mutants (R175H and R273H respectively) have no effect on expression from the *PLK1 *promoter, at least in the context of promoter-reporter (luciferase) assays [30]. The *TP53 *mutations identified in the present study show no common "hotspots" associated with PLK1 expression (data not shown). As expected, the most common types of the mutations in this cohort were missense mutations, clustered within exon 5 with a smaller group in exon 9 (Table 2). While there were insufficient cases in the cohort to provide unequivocal statistically-supported conclusions, there is a trend for the mutations in exon 5 to be associated with elevated PLK1 levels (in the tumours expressing high PLK1 levels, 6 mutations out of 12 in total are in exon 5 as compared with 11 in exon 5 out of 46 in total for those tumours expressing low or undetectable PLK1: Table 2). Exon 5 encodes amino acids 126 to 186 of p53. Future analyses should test this potential association in a significantly larger cohort. It will also be interesting to determine whether mutation of p53 within this region has any additional influence on PLK1 expression in cultured cells.

**Table 2:** Mutation spectra for the TP53 gene

Site of Mutation	Effect	PLK Neg (0-3)	PLK1 Pos (4-18)
**Exon 4**	Deletion	1	0
			
**Exon 5**	Missense	11	6
	Nonsense	1	0
			
**Exon 6**	Missense	5	0
	Nonsense	2	1
			
**Exon 7**	Deletion	7	2
			
**Exon 8**	Missense	9	2
			
**Exon 9**	Nonsense	1	0
			
**Exon 10**	Deletion	1	0
	Missense	2	0
			
**Intron 5**	Frame Shift	2	0
			
**Intron 6**	Frame Shift	1	1
			
**Intron 9**	Frame Shift	1	0
			
*Exon 5 and 6	Missense, Silent	1	0
			
*Exon 7, 8 and 9	Missense, Missense, Silent	1	0
			
		**46**	**12**

* Tumours where multiple mutations were present

An additional issue is that the association between *PLK1 *expression and *TP53 *mutational status is not absolute; for example, some tumours show elevated PLK1 levels yet retain wild type *TP53 *(Table 1). However, p53 function can be lost not only through mutation of the *TP53 *gene itself but also through mutation, or changes in the expression levels, of proteins that regulate the p53 response. For example, increased expression of the p53 inhibitors, MDM2 (see below) or MDM4, is associated with breast cancer development and is thought to suppress wild type p53 function [41,42]. It is, therefore, possible that such mechanisms may contribute to permitting PLK1 levels to rise in the tumours where mutation of the *TP53 *gene is absent. Consistent with this idea we also note a significant association between elevated levels of PLK1 and MDM2 expression (with potential p53 reduction) in our cohort as a whole (Table 1). However, owing to the small size of the subset that has both wild type *TP53 *and elevated PLK1, we cannot establish any significant association with MDM2 status. It is also possible that the overall association between MDM2 and PLK1 could reflect our previous observation that MDM2 levels may be regulated by PLK1 independently of p53 [30].

### Relationship of PLK1 expression to clinical outcome of breast cancer patients

**Figure 4:**
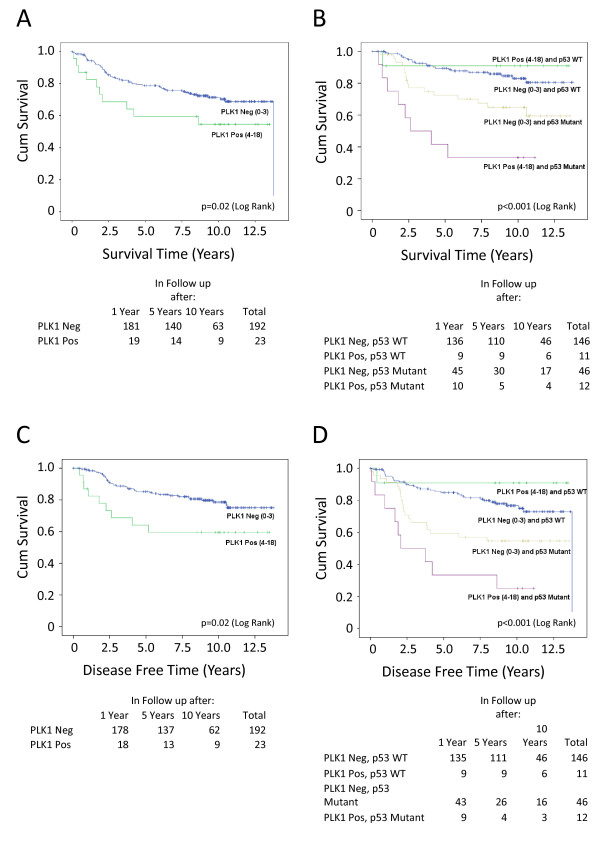
**Kaplan-Meier survival analysis**. **(A) **Data correlating PLK1 staining to breast cancer-specific survival in the cohort TMA24. *P *= 0.02. **(B) **Stratification of the data to show the influence of p53 status on the association between PLK1 staining and breast cancer-specific survival in the cohort TMA24. *P *< 0.001. **(C) **Data correlating PLK1 staining to disease free time in the cohort TMA24. *P *= 0.072 **(D) **Stratification of the data to show the influence of p53 status on the association between PLK1 staining and disease free time in the cohort TMA24. *P *< 0.001. The number of patients at 1-, 5- and 10-year time points are shown below each panel.

From a clinical perspective, the data show a striking association between elevated PLK1 levels in breast cancer cells and clinical outcome. PLK1 expression was associated almost exclusively with tumour grade, being present predominantly in grade 3 tumours. Moreover, the survival data (Table 1 and Figure 4A) clearly confirm that patients expressing elevated PLK1 expression show significantly reduced survival. These data are consistent with similar findings reported by others linking high levels of PLK1 to aggressive disease and poor outcome [17]. There are no significant associations between PLK1 expression and menopausal status, lymph node status or tumour size (Table 1).

By multivariate analysis, in the p53 mutant cancers, only PLK1 (*P *= 0.015) and the progesterone receptor (*P *= 0.04) were independently associated with survival. For p53 wild type cancers progesterone receptor (*P *= 0.041) and size (*P *= 0.007) were the only independent variables and for all patients (no discrimination by mutation status), progesterone receptor (*P *< 0.001), tumour grade (*P *= 0.023), node positivity (*P *= 0.011) and size (*P *= 0.031) were independently associated with survival.

There are two additional associations of particular interest and novelty. Firstly, the occurrence of elevated PLK1 levels together with *TP53 *mutation is associated with poor outcome (Figure 4B), much more so than in patients harbouring *TP53 *mutation alone. Interestingly, patients with tumours that show elevated PLK1 expression, but retain wild type *TP53*, have a relatively favourable outcome (Figure 4B). This observation indicates that changes in PLK1 levels may also occur independently of regulation by p53 (that is, through changes to an independent PLK1 regulatory pathway). It also suggests very strongly that p53 is protective against dysregulated PLK1, (which is presumably dysregulated through p53-independent mechanisms), in the context of human disease development. The second interesting feature is the close association of elevated PLK1 with the triple negative phenotype (Table 1). Triple negative tumours tend to have mutation(s) of the *TP53 *gene and are the most difficult to treat. These data again raise the possibility that tumours of this phenotype, expressing elevated PLK1 and harbouring *TP53 *mutation, may benefit from the use of novel PLK1 inhibitors [7].

**Figure 5:**
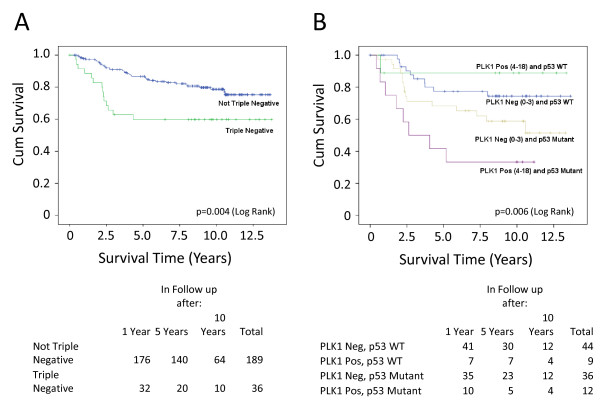
**Kaplan-Meier survival analysis of patients with Grade 3 tumours**. **(A) **Data correlating PLK1 staining to breast cancer-specific survival. *P *= 0.004. **(B) **Stratification of the data to show the influence of p53 status on the association between PLK1 staining and breast cancer-specific survival. *P *= 0.006. The number of patients at 1-, 5- and 10-year time points are shown below each panel.

The finding that PLK1 expression is associated almost exclusively with tumour grade (grade 3) could suggest that PLK1 is a passive feature of grade 3 tumours as opposed to a change that may drive tumour development. To address this issue, grade 3 tumours were re-examined and demonstrate clearly that PLK1 status was strongly associated with outcome within the grade 3 tumour subgroup (Figure 5A). This underscores the adverse influence on survival of having both PLK1 over-expression and mutant *TP53 *status within these high grade cancers (Figure 5B).

## Conclusions

Immunohistochemical analysis and DNA sequencing analysis of 215 primary breast tumours have demonstrated a striking association between PLK1 expression and *TP53 *gene mutation. These observations are consistent with our recently-published molecular model in which wild type functional p53 behaves as an important transcriptional repressor of PLK1. Targeting PLK1 in p53 mutant breast cancer, including poor prognosis, triple negative breast cancer, may offer therapeutic opportunities.

## Abbreviations

CSV: Comma Separated Values; ERα: estrogen receptor α; HER-2: human epidermal growth factor receptor-2; MDM2: murine double minute, clone 2; MDM4: murine double minute: clone 4; PLK1: polo-like kinase-1; PR: progesterone receptor; TBS: Tris-buffered saline; TMA: tumour microarray; *TP53: *tumour antigen p53 gene.

## Competing interests

The authors declare that they have no competing interests.

## Authors' contributions

SIK carried out the experiments and data analysis, interpreted the data and wrote drafts of the manuscript. SB carried out some of the experimental studies and data analysis. PRQ generated the database and carried out the computer-based analysis and statistical application. LJ and CAP undertook all of the pathological analysis and interpretation. AMT conceived and designed the studies, and analysed and interpreted the data. DWM conceived and designed the studies, analysed and interpreted the data, and wrote the final manuscript. All authors read, made suggestions and approved the final manuscript.

## References

[B1] Riley T Sontag E Chen P Levine A Transcriptional control of human p53-regulated genes Nat Rev Mol Cell Biol 2008 9 402 412 10.1038/nrm2395 18431400

[B2] Vousden KH Lane DP p53 in health and disease Nat Rev Mol Cell Biol 2007 8 275 283 10.1038/nrm2147 17380161

[B3] Vousden KH Prives C Blinded by the light: the growing complexity of p53 Cell 2009 137 413 431 10.1016/j.cell.2009.04.037 19410540

[B4] Goh AM Coffill CR Lane DP The role of mutant p53 in human cancer J Pathol 2010 223 116 126 21125670 10.1002/path.2784

[B5] Bonnefoi H Piccart M Bogaerts J Mauriac L Fumoleau P Brain E Petit T Rouanet P Jassem J Blot E Zaman K Cufer T Lortholary A Lidbrink E Andre S Litiere S Lago LD Becette V Cameron DA Bergh J Iggo R TP53 status for prediction of sensitivity to taxane versus non-taxane neoadjuvant chemotherapy in breast cancer (EORTC 10994/BIG 1-00): a randomised phase 3 trial Lancet Oncol 2011 12 527 539 10.1016/S1470-2045(11)70094-8 21570352 PMC4172919

[B6] Archambault V Glover DM Polo-like kinases: conservation and divergence in their functions and regulation Nat Rev Mol Cell Biol 2009 10 265 275 10.1038/nrm2653 19305416

[B7] Strebhardt K Multifaceted polo-like kinases: drug targets and antitargets for cancer therapy Nat Rev Drug Discov 2010 9 643 660 10.1038/nrd3184 20671765

[B8] Strebhardt K Ullrich A Targeting polo-like kinase 1 for cancer therapy Nat Rev Cancer 2006 6 321 330 10.1038/nrc1841 16557283

[B9] Smith MR Wilson ML Hamanaka R Chase D Kung H Longo DL Ferris DK Malignant transformation of mammalian cells initiated by constitutive expression of the polo-like kinase Biochem Biophys Res Commun 1997 234 397 405 10.1006/bbrc.1997.6633 9177283

[B10] Dietzmann K Kirches E von Bossanyi P Jachau K Mawrin C Increased human polo-like kinase-1 expression in gliomas J Neuro-oncol 2001 53 1 11 10.1023/a:1011808200978 11678424

[B11] Ito Y Miyoshi E Sasaki N Kakudo K Yoshida H Tomoda C Uruno T Takamura Y Miya A Kobayashi K Matsuzuka F Matsuura N Kuma K Miyauchi A Polo-like kinase 1 overexpression is an early event in the progression of papillary carcinoma Br J Cancer 2004 90 414 418 10.1038/sj.bjc.6601540 14735186 PMC2409566

[B12] Knecht R Elez R Oechler M Solbach C von Ilberg C Strebhardt K Prognostic significance of polo-like kinase (PLK) expression in squamous cell carcinomas of the head and neck Cancer Res 1999 59 2794 2797 10383133

[B13] Kneisel L Strebhardt K Bernd A Wolter M Binder A Kaufmann R Expression of polo-like kinase (PLK1) in thin melanomas: a novel marker of metastatic disease J Cutan Pathol 2002 29 354 358 10.1034/j.1600-0560.2002.290605.x 12135466

[B14] Takahashi T Sano B Nagata T Kato H Sugiyama Y Kunieda K Kimura M Okano Y Saji S Polo-like kinase 1 (PLK1) is overexpressed in primary colorectal cancers Cancer Sci 2003 94 148 152 10.1111/j.1349-7006.2003.tb01411.x 12708489 PMC11160284

[B15] Tokumitsu Y Mori M Tanaka S Akazawa K Nakano S Niho Y Prognostic significance of polo-like kinase expression in esophageal carcinoma Int J Oncol 1999 15 687 692 10493949 10.3892/ijo.15.4.687

[B16] Weichert W Denkert C Schmidt M Gekeler V Wolf G Kobel M Dietel M Hauptmann S Polo-like kinase isoform expression is a prognostic factor in ovarian carcinoma Br J Cancer 2004 90 815 821 10.1038/sj.bjc.6601610 14970859 PMC2410182

[B17] Weichert W Kristiansen G Winzer KJ Schmidt M Gekeler V Noske A Muller BM Niesporek S Dietel M Denkert C Polo-like kinase isoforms in breast cancer: expression patterns and prognostic implications Virchows Arch 2005 446 442 450 10.1007/s00428-005-1212-8 15785925

[B18] Weichert W Schmidt M Gekeler V Denkert C Stephan C Jung K Loening S Dietel M Kristiansen G Polo-like kinase 1 is overexpressed in prostate cancer and linked to higher tumor grades Prostate 2004 60 240 245 10.1002/pros.20050 15176053

[B19] Wolf G Elez R Doermer A Holtrich U Ackermann H Stutte HJ Altmannsberger HM Rubsamen-Waigmann H Strebhardt K Prognostic significance of polo-like kinase (PLK) expression in non-small cell lung cancer Oncogene 1997 14 543 549 10.1038/sj.onc.1200862 9053852

[B20] Yamada S Ohira M Horie H Ando K Takayasu H Suzuki Y Sugano S Hirata T Goto T Matsunaga T Hiyama E Hayashi Y Ando H Suita S Kaneko M Sasaki F Hashizume K Ohnuma N Nakagawara A Expression profiling and differential screening between hepatoblastomas and the corresponding normal livers: identification of high expression of the PLK1 oncogene as a poor-prognostic indicator of hepatoblastomas Oncogene 2004 23 5901 5911 10.1038/sj.onc.1207782 15221005

[B21] Wolf G Hildenbrand R Schwar C Grobholz R Kaufmann M Stutte HJ Strebhardt K Bleyl U Polo-like kinase: a novel marker of proliferation: correlation with estrogen-receptor expression in human breast cancer Pathol Res Pract 2000 196 753 759 11186170 10.1016/S0344-0338(00)80107-7

[B22] Ando K Ozaki T Yamamoto H Furuya K Hosoda M Hayashi S Fukuzawa M Nakagawara A Polo-like kinase 1 (Plk1) inhibits p53 function by physical interaction and phosphorylation J Biol Chem 2004 279 25549 25561 10.1074/jbc.M314182200 15024021

[B23] Incassati A Patel D McCance DJ Induction of tetraploidy through loss of p53 and upregulation of Plk1 by human papillomavirus type-16 E6 Oncogene 2006 25 2444 2451 10.1038/sj.onc.1209276 16369493

[B24] Kho PS Wang Z Zhuang L Li Y Chew JL Ng HH Liu ET Yu Q p53-regulated transcriptional program associated with genotoxic stress-induced apoptosis J Biol Chem 2004 279 21183 21192 10.1074/jbc.M311912200 15016801

[B25] Smits VA Klompmaker R Arnaud L Rijksen G Nigg EA Medema RH Polo-like kinase 1 is a target of the DNA damage checkpoint Nat Cell Biol 2000 2 672 676 10.1038/35023629 10980711

[B26] Sur S Pagliarini R Bunz F Rago C Diaz LA Jr Kinzler KW Vogelstein B Papadopoulos N A panel of isogenic human cancer cells suggests a therapeutic approach for cancers with inactivated p53 Proc Natl Acad Sci USA 2009 106 3964 3969 10.1073/pnas.0813333106 19225112 PMC2656188

[B27] van Vugt MA Medema RH Getting in and out of mitosis with Polo-like kinase-1 Oncogene 2005 24 2844 2859 10.1038/sj.onc.1208617 15838519

[B28] van Vugt MA Smits VA Klompmaker R Medema RH Inhibition of polo-like kinase-1 by DNA damage occurs in an ATM- or ATR-dependent fashion J Biol Chem 2001 276 41656 41660 10.1074/jbc.M101831200 11514540

[B29] Zhu H Chang BD Uchiumi T Roninson IB Identification of promoter elements responsible for transcriptional inhibition of polo-like kinase 1 and topoisomerase IIalpha genes by p21(WAF1/CIP1/SDI1) Cell Cycle 2002 1 59 66 12429910

[B30] McKenzie L King S Marcar L Nicol S Dias SS Schumm K Robertson P Bourdon JC Perkins N Fuller-Pace F Meek DW p53-dependent repression of polo-like kinase-1 (PLK1) Cell Cycle 9 4200 4212 20962589 10.4161/cc.9.20.13532 PMC3055203

[B31] Hadad SM Baker L Quinlan PR Robertson KE Bray SE Thomson G Kellock D Jordan LB Purdie CA Hardie DG Fleming S Thompson AM Histological evaluation of AMPK signalling in primary breast cancer BMC Cancer 2009 9 307 10.1186/1471-2407-9-307 19723334 PMC2744705

[B32] Baker L Quinlan PR Patten N Ashfield A Birse-Stewart-Bell LJ McCowan C Bourdon JC Purdie CA Jordan LB Dewar JA Wu L Thompson AM p53 mutation, deprivation and poor prognosis in primary breast cancer Br J Cancer 2010 102 719 726 10.1038/sj.bjc.6605540 20104224 PMC2837559

[B33] Detre S Saclani Jotti G Dowsett M A "quickscore" method for immunohistochemical semiquantitation: validation for oestrogen receptor in breast carcinomas J Clin Pathol 1995 48 876 878 10.1136/jcp.48.9.876 7490328 PMC502883

[B34] Quinlan PR Reed C Thompson A INSPIRE: an integrated agent based system for hypothesis generation within cancer datasets WI-IAT 2008 3 587 590 2008; IEEE/WIC/ACM International Conference on Web Intelligence and Intelligent Agent Technology: 9-12 December 2008

[B35] Yuan J Horlin A Hock B Stutte HJ Rubsamen-Waigmann H Strebhardt K Polo-like kinase, a novel marker for cellular proliferation Am J Pathol 1997 150 1165 1172 9094972 PMC1858156

[B36] Golsteyn RM Schultz SJ Bartek J Ziemiecki A Ried T Nigg EA Cell cycle analysis and chromosomal localization of human Plk1, a putative homologue of the mitotic kinases Drosophila polo and Saccharomyces cerevisiae Cdc5 J Cell Sci 1994 107 1509 1517 7962193 10.1242/jcs.107.6.1509

[B37] Hamanaka R Smith MR O'Connor PM Maloid S Mihalic K Spivak JL Longo DL Ferris DK Polo-like kinase is a cell cycle-regulated kinase activated during mitosis J Biol Chem 1995 270 21086 21091 10.1074/jbc.270.36.21086 7673138

[B38] Lee KS Yuan YL Kuriyama R Erikson RL Plk is an M-phase-specific protein kinase and interacts with a kinesin-like protein, CHO1/MKLP-1 Mol Cell Biol 1995 15 7143 7151 8524282 10.1128/mcb.15.12.7143 PMC230970

[B39] Uchiumi T Longo DL Ferris DK Cell cycle regulation of the human polo-like kinase (PLK) promoter J Biol Chem 1997 272 9166 9174 10.1074/jbc.272.14.9166 9083047

[B40] Yim H Erikson RL Polo-like kinase 1 depletion induces DNA damage in early S prior to caspase activation Mol Cell Biol 2009 29 2609 2621 10.1128/MCB.01277-08 19289504 PMC2682042

[B41] Danovi D Meulmeester E Pasini D Migliorini D Capra M Frenk R de Graaf P Francoz S Gasparini P Gobbi A Helin K Pelicci PG Jochemsen AG Marine JC Amplification of MDMx (or MDM4) directly contributes to tumor formation by inhibiting p53 tumor suppressor activity Mol Cell Biol 2004 24 5835 5843 10.1128/MCB.24.13.5835-5843.2004 15199139 PMC480894

[B42] Onel K Cordon-Cardo C MDM2 and prognosis Mol Cancer Res 2004 2 1 8 14757840

